# Hypoxia-induced exosome secretion promotes survival of African-American and Caucasian prostate cancer cells

**DOI:** 10.1038/s41598-018-22068-4

**Published:** 2018-03-01

**Authors:** Gati K. Panigrahi, Prakash P. Praharaj, Taylor C. Peak, Jessica Long, Ravi Singh, Johng S. Rhim, Zakaria Y. Abd Elmageed, Gagan Deep

**Affiliations:** 1 Department of Cancer Biology, Wake Forest School of Medicine, Winston-Salem, North Carolina USA; 2 Wake Forest Baptist Comprehensive Cancer Center, Wake Forest School of Medicine, Winston-Salem, North Carolina USA; 3 Center for Prostate Disease Research, Department of Surgery, Uniformed Services University of Health Sciences, Bethesda, MD USA; 4 Department of Pharmaceutical Sciences, Texas A&M Rangel College of Pharmacy, College Station, Texas USA

## Abstract

African American men in the United States have higher mortality due to prostate cancer (PCa) compared to other races. One reason for this disparity is the lack of in-depth understanding of the PCa biology in African Americans. For example, hypoxia in prostate tumor microenvironment is associated with adverse prognosis; still, no hypoxia-related studies have been reported in African Americans. Here, we compared African-American and Caucasian PCa cells for exosome secretion under normoxic (21% O_2_) and hypoxic (1% O_2_) conditions. All cell lines showed higher exosome secretion under hypoxia but it was clearly more prominent in African-American PCa cells. Further, under hypoxia, Rab5 (a biomarker for early endosome) was clustered in perinuclear region; and CD63 (a biomarker for exosomes and multivesicular endosomes) showed greater co-localization with actin cytoskeleton especially in African American PCa cells. Importantly, exosome biogenesis inhibitors GW4869 (10–20 µM) or DMA (10–20 µg/ml) significantly decreased cell viability and clonogenicity in PCa cells. Interestingly, we also observed higher level of lactic acid loaded in exosomes secreted under hypoxia. Overall, under chronic hypoxia, PCa cells secrete more exosomes as a survival mechanism to remove metabolic waste.

## Introduction

African American men in the United States have higher incidence and death rates from prostate cancer (PCa) than men of other races. In 2016 alone, about 30,000 cases of PCa were diagnosed and about 4,450 PCa deaths were reported among African American men^1^. In addition, African American men are more frequently diagnosed with advanced PCa at earlier ages, with worse prognoses and lower overall survival rates than Caucasian men^1–5^. Currently, we have only limited knowledge about key factors and molecular pathways that drive PCa in African Americans; and most diagnostic and treatment decisions are based on studies performed in Caucasian PCa cells or patients. These treatments might not be equally efficacious in African Americans; therefore, further research into the unique tumor biology of PCa in African Americans is urgently needed to improve their prognosis and treatment.

Hypoxia (low oxygen conditions) in prostate tumors is an early event associated with an aggressive phenotype^6–8^. Hypoxic conditions promote genetic, metabolic, and proteomic changes leading to increased glycolysis, angiogenesis, survival, stemness, invasiveness, and selection of resistant clones^6,9,10^. PCa hypoxia status and/or expression of hypoxia-induced signaling molecules are associated with poor prognosis and treatment failure^11–15^. For example, in 100 human primary prostate tumors, HIF-1α expression was associated with treatment failure, disease relapse, and decreased metastasis-free survival and castration-resistant prostate cancer (CRPC)-free survival^11^. More importantly, prostate tumors lacking HIF-1α expression did not metastasize or develop CRPC^11^.

Several clinical studies have reported that hypoxia in primary PCa is linked with disease progression, disease recurrence, and treatment failure, whether the treatment was surgery, radiation therapy, or hormone therapy^12–14,16–19^. On the contrary, men who regularly used digoxin (a non-specific HIF-1α inhibitor) showed a 25% decrease in risk of developing PCa, including potentially lethal disease^20^. In another report, use of nonspecific HIF-1α inhibitors in patients with PCa improved progression-free survival time and reduced the risk of developing CRPC and metastasis^21^. Therefore, there is good evidence that hypoxia and activation of hypoxia-related signaling pathways in prostate tumors determine growth, promotion, metastasis, hormone-refractory progression, and treatment outcome.

Exosomes are approximately 30–150 nm in diameter released by all cell types, and are present in most biological fluids^22^. These vesicles are now called extracellular vesicles (EVs), and originate intracellularly in endosomes. Recently, we characterized exosomes secreted by Caucasian PCa cells under hypoxic conditions^23,24^. These studies showed that hypoxic PCa exosomes are loaded with a higher number of unique proteins and triglycerides compared to normoxic PCa exosomes; and hypoxic PCa exosomes promoted the epithelial-mesenchymal transition (EMT) and stemness in naïve PCa cells. Several other studies have also reported the key role of exosomes secreted by cancer cells under hypoxia in remodeling the tumor microenvironment and disease progression^23,25^; however, no such study has been performed to delineate racial differences in exosomes secretion under hypoxic conditions.

All our existing knowledge about hypoxia-induced signaling and biological effects is based on PCa cells from Caucasian patients. Despite the key role of hypoxia in prostate carcinogenesis, not even a single hypoxia-related study has been reported in African American PCa cells. In the present study, we characterized African American PCa cells under hypoxia in terms of their ability to secrete exosomes. Results suggest that chronic hypoxia promote exosome secretion and offer a survival advantage to cells, probably via removal of metabolic waste.

## Results

### Hypoxia promotes exosome secretion in PCa cells

**Figure 1:**
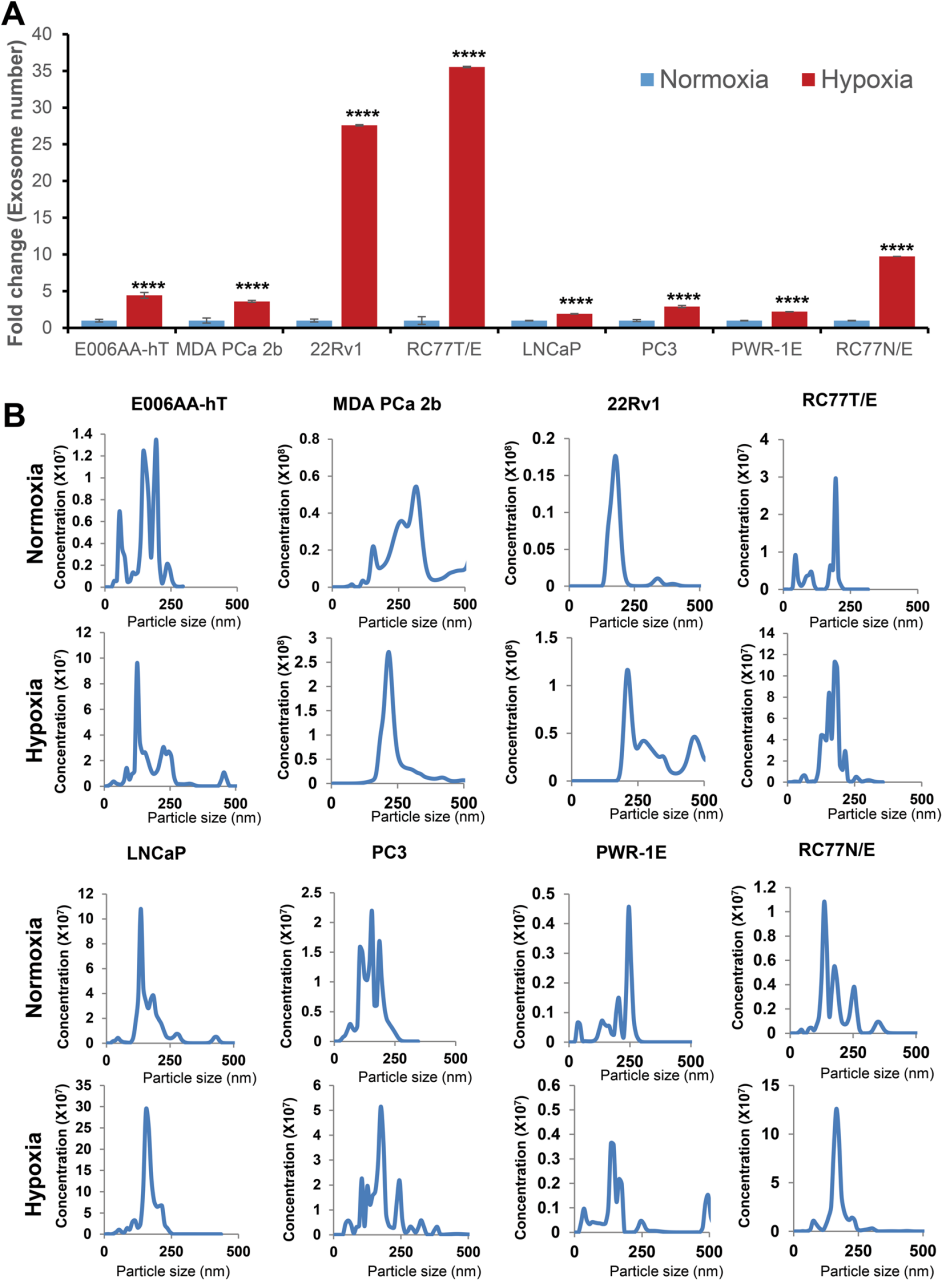
Hypoxia promotes exosome secretion in PCa cells. (**A**) Each cell line was plated (1 × 10^6^ cells in T75 flask) in complete media; and then cultured for 48 hrs in exosome-depleted media under normoxic (21% O_2_) or hypoxic (1% O_2_) conditions. Thereafter, conditioned media was collected and exosomes isolated by ultracentrifugation method as detailed in the methods. Exosome concentration and size distribution was measured by NTA. In each case, exosome concentration was normalized with corresponding cell numbers and presented as fold change (normoxia versus hypoxia) for all cell lines. Data represent mean ± SE of five videos. ****p < 0.0001. (**B**) Representative particle distributions for normoxic and hypoxic exosomes from all cell lines are presented.

All cell lines tested showed increased concentrations of exosomes under hypoxia compared to normoxia (Fig. 1A). The increase under hypoxia with respect to normoxia in E006AA-hT, MDA PCa 2b, 22Rv1, RC77T/E, LNCaP, PC3, PWR1E, and RC77N/E cells was 4.4 fold, 3.6 fold, 27.5 fold, 35.5 fold, 1.9 fold, 2.9 fold, 2.2 fold and 9.7 fold, respectively (Fig. 1A). Representative distributions of exosome size and concentration are shown in Fig. 1B.

**Figure 2:**
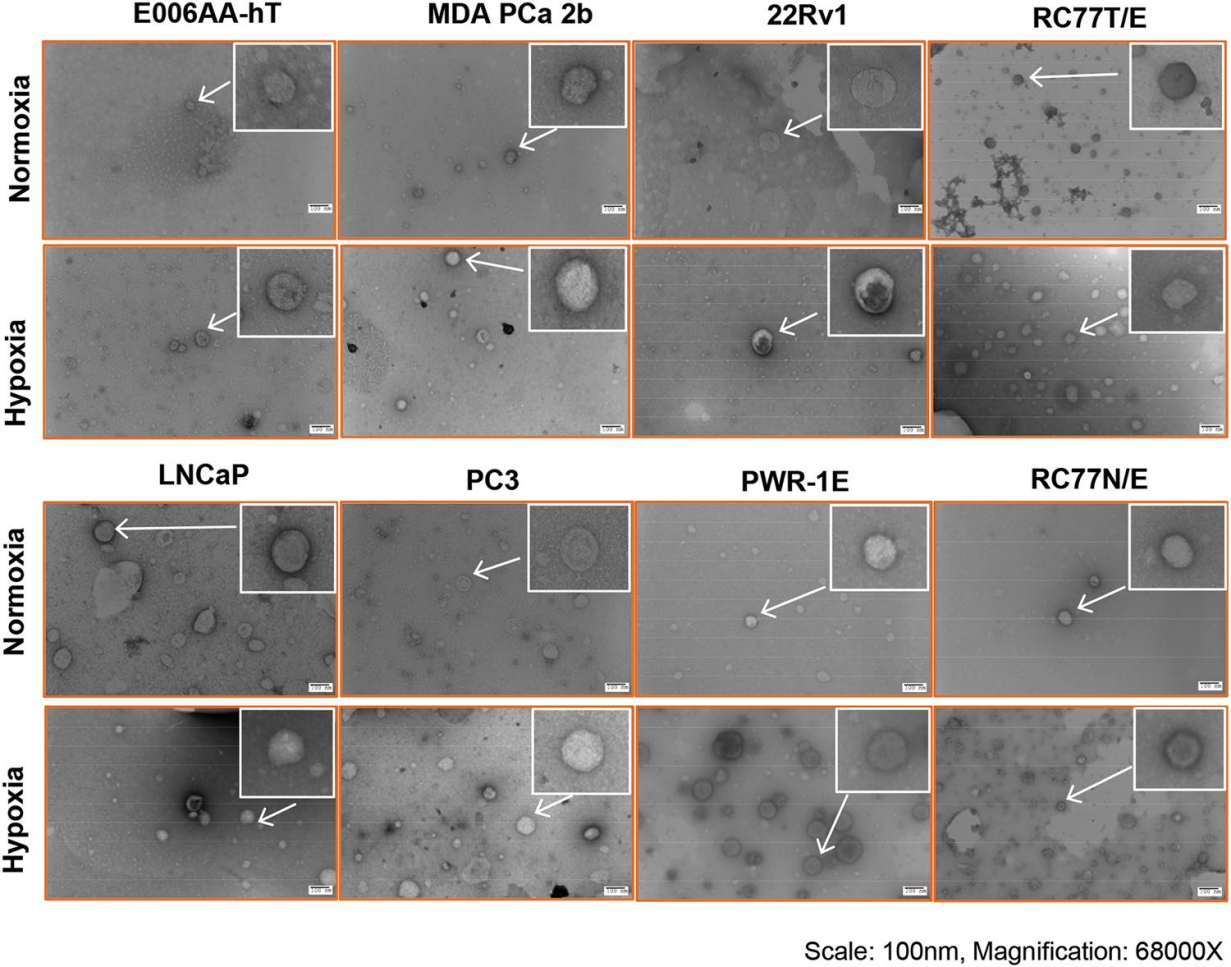
Characterization of exosomes by electron microscopy. Exosomes were processed for transmission electron microscopy as detailed in methods. Representative electron microscope images are shown (Scale-100 nm, Magnification = 68000×). Inset shows a magnified portion of the picture.

Next, we characterized the exosomes isolated from PCa cells cultured under normoxic and hypoxic conditions by TEM. As shown in Fig. 2, in all the cell lines, exosome size was ≤100 nm.

### Hypoxia promotes early endosome formation and fusion of multivesicular endosomes (MVE) with plasma membrane

**Figure 3:**
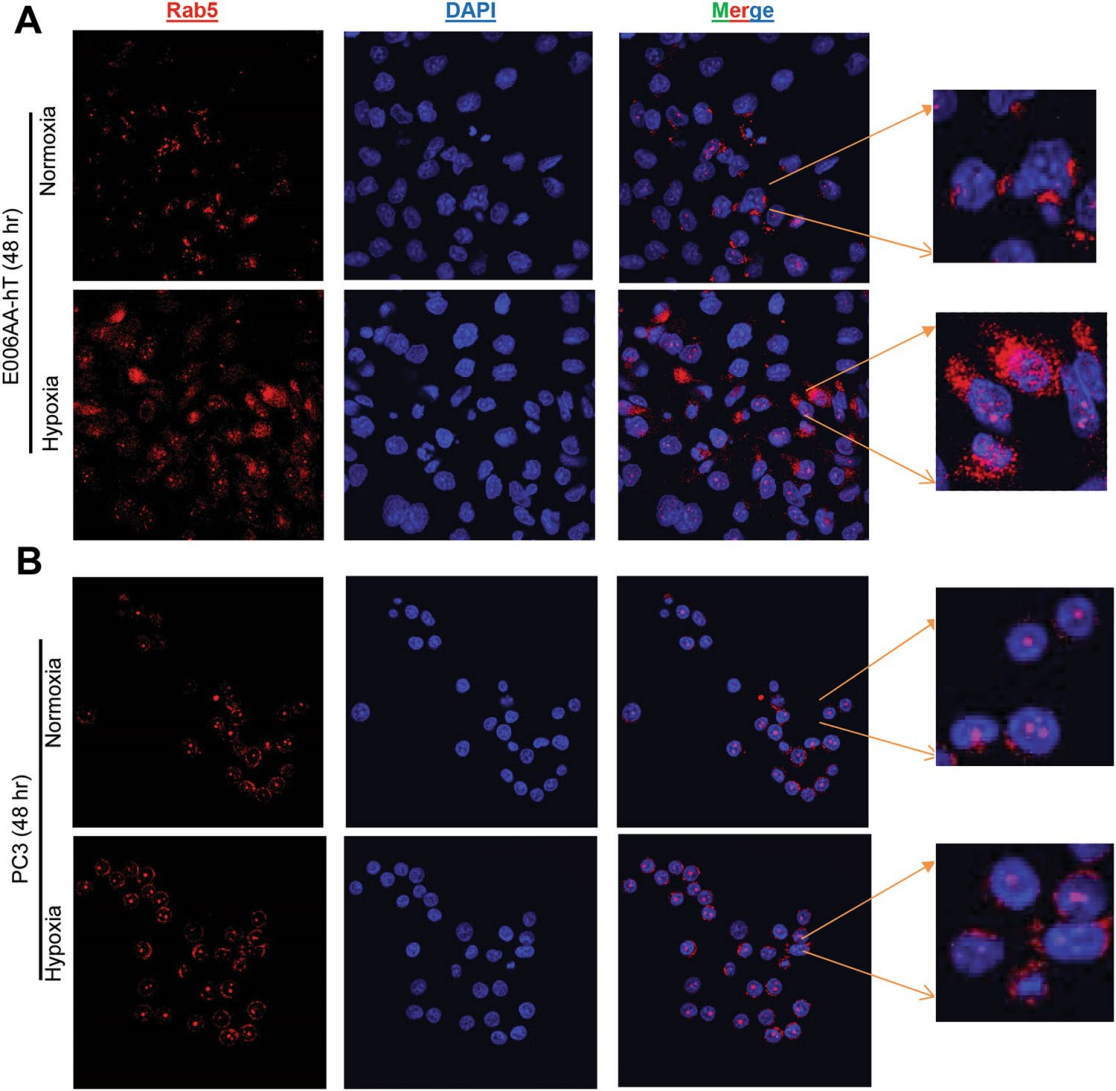
Effect of hypoxia on RAB5 in E006AA-hT and PC3 cells. E006AA-hT (**A**) and PC3 cells (**B**) were cultured under normoxic (21% O_2_) or hypoxic (1% O_2_) conditions for 48 hrs and processed for Rab5 staining  (red) by confocal microscopy. DAPI (blue) was used to stain nuclei. Merge images show the overlay of red and blue colors. Inset show a magnified portion of the image. Representative images are shown at 600x.

To understand why exosome secretion was higher in PCa cells under hypoxia, we analyzed formation of early endosomes and MVEs by staining for Rab5 and CD63 in African American PCa E006AA-hT and Caucasian PCa PC3 cells, respectively. Rab5 belongs to a family of small GTPases that regulates various steps in vesicular trafficking^26^. Rab5 regulates clathrin-coated vesicle-mediated transport from the cell membrane to early endosomes and homotypic early endosome fusion^27^. In both PCa cell lines under hypoxia, Rab5 was more clustered in the perinuclear region especially in E006AA-hT cells (Fig. 3A,B).

**Figure 4:**
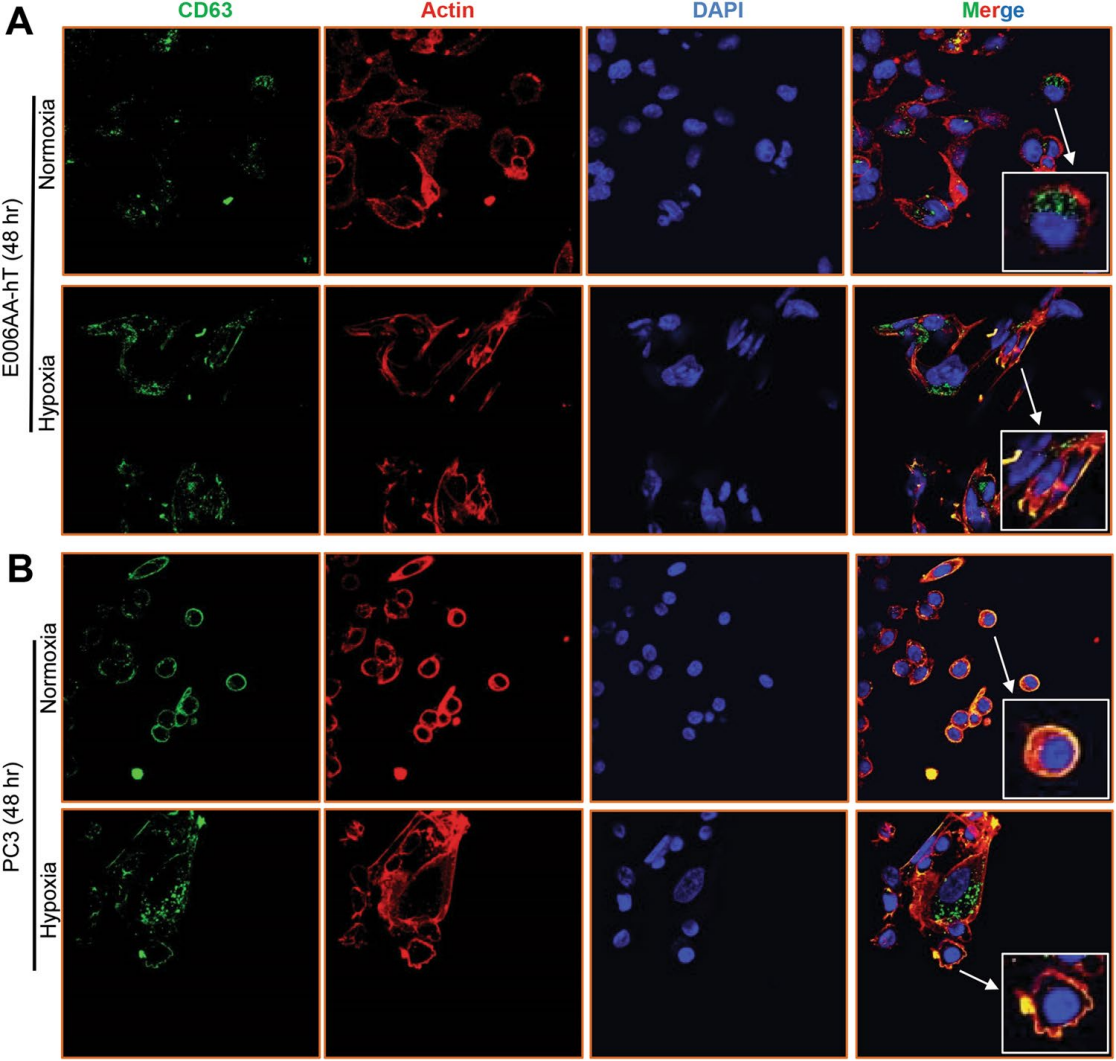
Effect of hypoxia on the CD63 and actin co-localization in E006AA-hT and PC3 cells. E006AA-hT (**A**) and PC3 cells (**B**) were cultured under normoxic (21% O_2_) or hypoxic (1% O_2_) conditions for 48 hrs and processed for CD63 (green), actin (red), nuclei (DAPI, blue) staining  by confocal microscopy. Merge images show the overlay of green, red and blue. Inset show a magnified portion of the image. Representative images are shown at 600x.

CD63 is a member of the tetraspanin family and mostly used as a biomarker for exosomes. CD63 is also present on MVEs; therefore, we next examined CD63 expression in E006AA-hT and PC3 cells under normoxia and hypoxia. As shown in Fig. 4A, CD63 level was higher in E006AA-hT cells under hypoxia compared to normoxia. In PC3 cells there was no obvious change in CD63 level under hypoxia (Fig. 4B).

During exosome biogenesis MVEs move along the cytoskeleton and fuse with plasma membranes. Specific actin structures such as invadopodia are important in MVEs fusion and exosome release^28,29^. To explore the potential role of actin remodeling under hypoxia with respect to exosome biogenesis, we also analyzed actin expression using confocal microscopy. E006AA-hT cells showed more organized actin, and a greater colocalization of actin with CD63 under hypoxia compared to normoxia (Fig. 4A). In PC3 cells, quite similar colocalization of CD63 and actin was observed under both normoxia and hypoxia (Fig. 4B). Greater colocalization of MVEs with the actin cytoskeleton could explain relatively higher exosome secretion in E006AA-hT cells under hypoxia compared to PC3 cells.

### Inhibition of exosome biogenesis reduces growth in PCa cells

**Figure 5:**
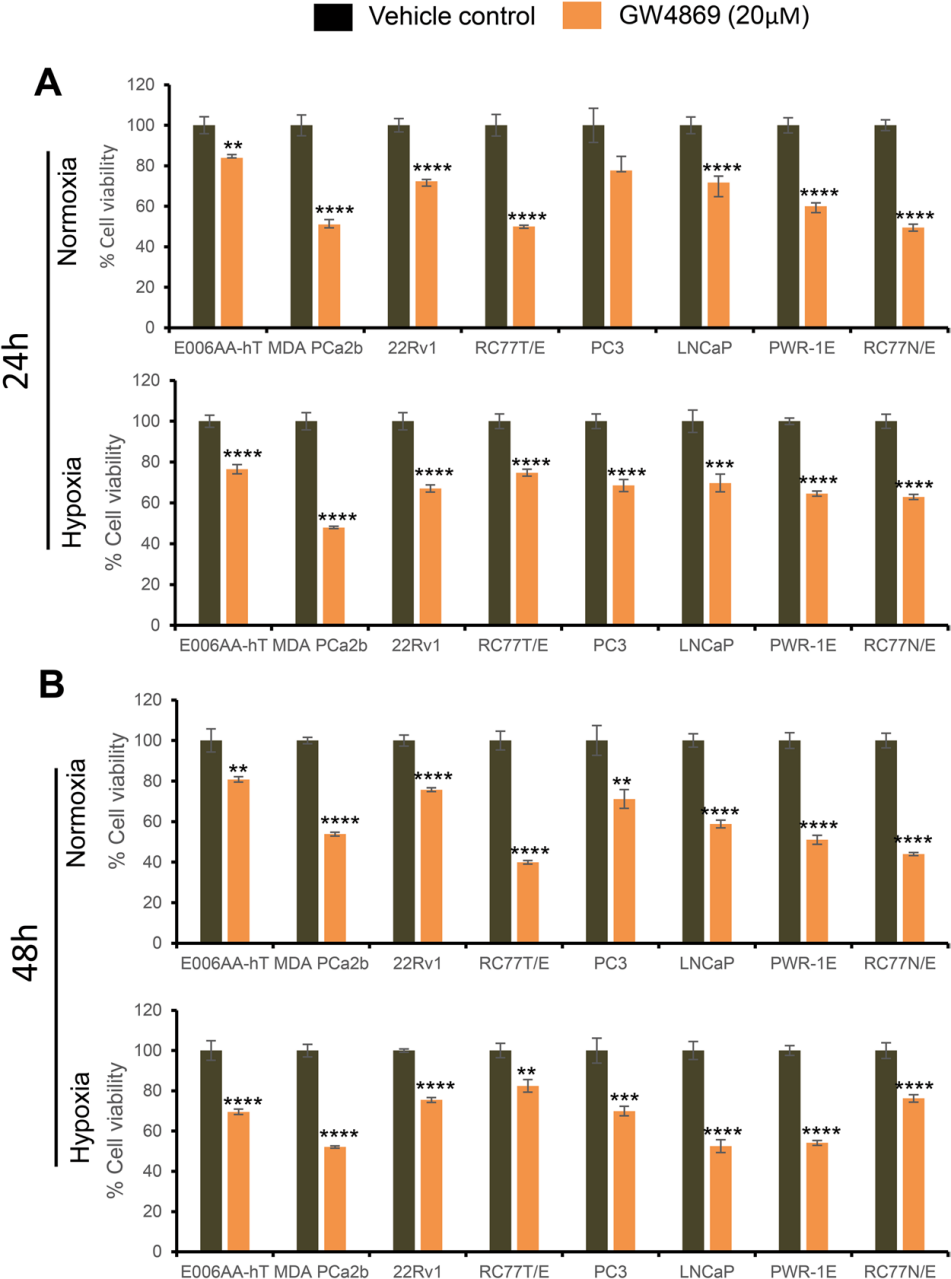
GW4869 inhibits exosome biogenesis by decreasing cell viability under both normoxic and hypoxic conditions. (**A**,**B**) All cell lines were plated (2,000 cells per well) in 96-well plates, treated with GW4869 (20 µM), and cultured under normoxic (21% O_2_) or hypoxic (1% O_2_) conditions for 24 hrs and 48 hrs. At the end, cell viability was measured by MTT assay. Data are presented as mean ± SE of five replicates with control set at 100%. **p < 0.01, ***p < 0.001, ****p < 0.0001.

Next, to determine whether exosome secretion is helpful in cell survival, we treated all cell lines with an exosome biogenesis inhibitor, GW4869, and assessed their viability under normoxic and hypoxic conditions. Cell viability decreased significantly following GW4869 treatment in all cell lines at both time points (24 and 48 h), under both normoxia and hypoxia (Fig. 5).

**Figure 6:**
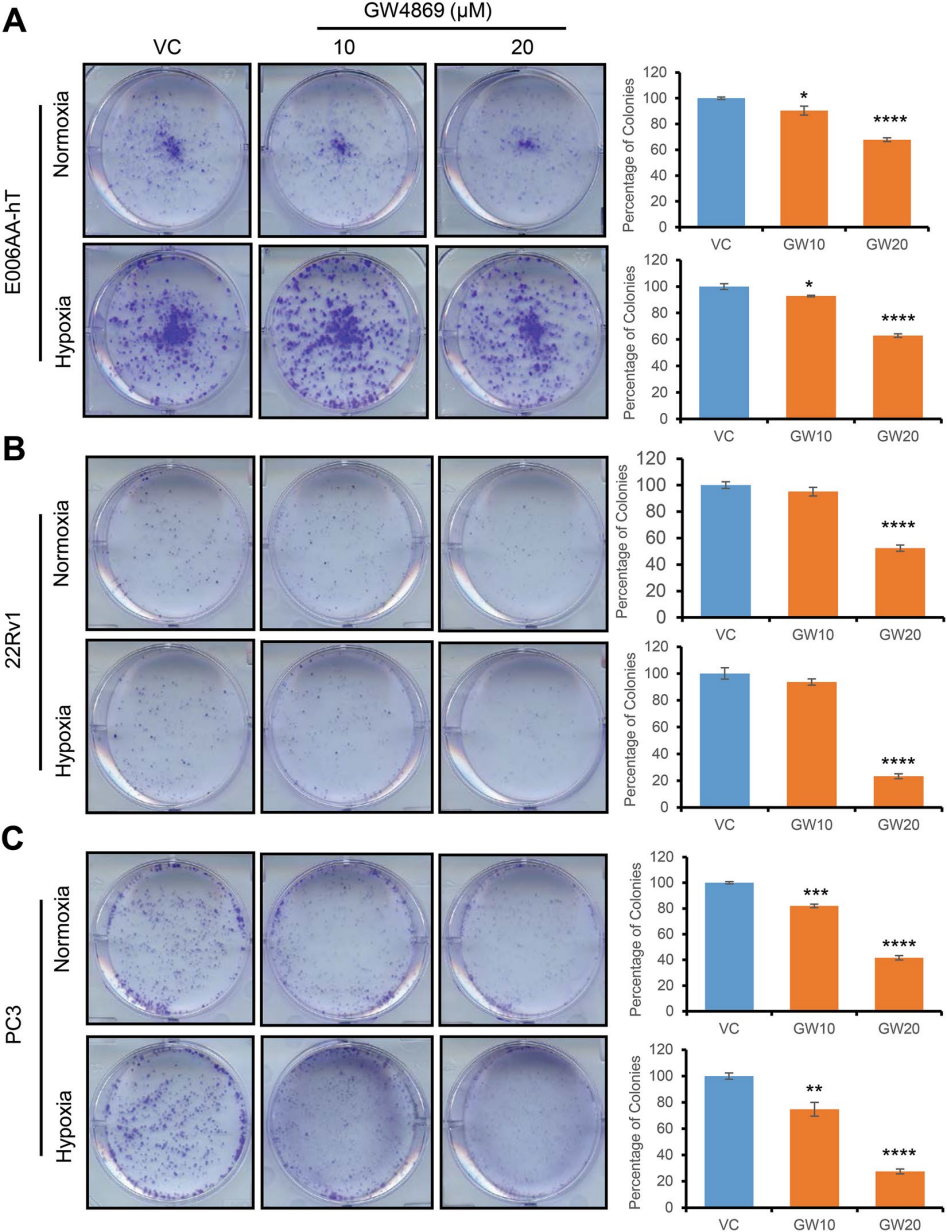
GW4869 reduces clonogenicity of PCa cells. (**A**–**C**) E006AA-hT, 22Rv1, and PC3 cells (1,000 cells per well) were cultured overnight in 6-well plates, and then treated once with DMSO (control) or GW4869 (10–20 μM) and cultured under normoxic (21% O_2_) or hypoxic (1% O_2_) conditions for 7-8 days. At the end, cells were processed and colony number was counted as detailed in methods. Representative images are presented and data is presented as mean ± SE (n = 3). *p < 0.05, **p < 0.01, ***p < 0.001, ****p < 0.0001.

**Figure 7:**
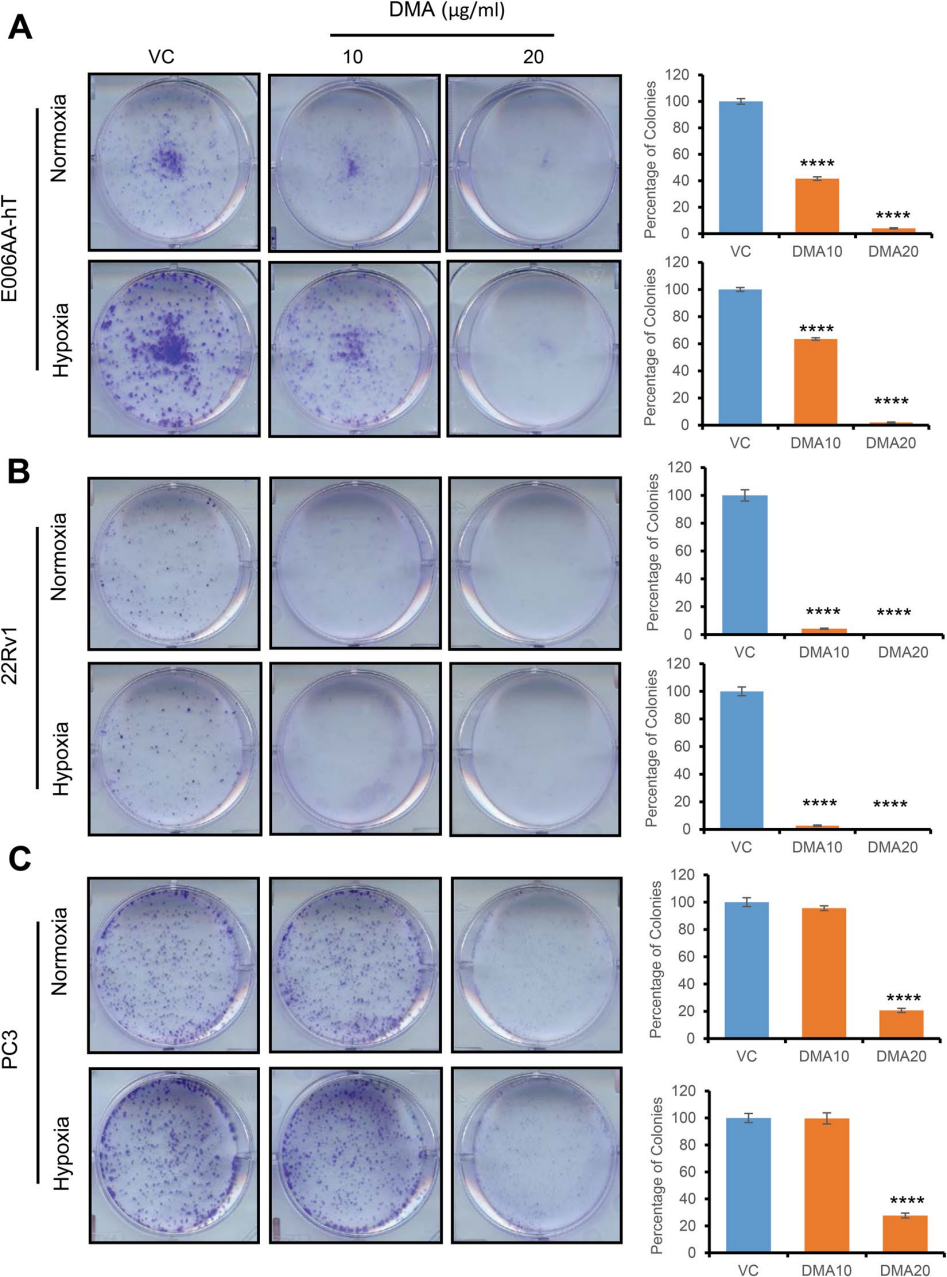
DMA inhibits clonogenicity of PCa cells. (**A**–**C**) E006AA-hT, 22Rv1 and PC3 cells (1,000 cells per well) were cultured overnight in 6-well plates, and then treated once with DMSO (control) or DMA (10–20 μg/ml) and cultured under normoxic (21% O_2_) or hypoxic (1% O_2_) conditions for 7–8 days. At the end, cells were processed and colony number was counted as detailed in methods. Representative images are presented and data is presented as mean ± SE (n = 3). ****p < 0.0001.

Next, we performed clonogenic assays to determine the effects of GW4869 on colony-forming potential of PCa cells (E006AA-hT, 22Rv1, and PC3). GW4869 (10–20 µg/ml) treatment reduced the clonogenicity of all the cell lines (Fig. 6A–C). To further validate these results, we treated these cell lines with another exosome biogenesis inhibitor, DMA (10–20 µg/ml). Treatment strongly inhibited clonogenicity of all three cell lines under both normoxic and hypoxic conditions (Fig. 7A–C).

Taken together, the MTT and clonogenic assay results confirmed that exosome secretion by PCa cells is a potential survival mechanism, and that inhibition of exosome biogenesis reduces cancer cell growth.

### Hypoxic PCa cells secrete exosomes loaded with higher amounts of lactate

**Figure 8:**
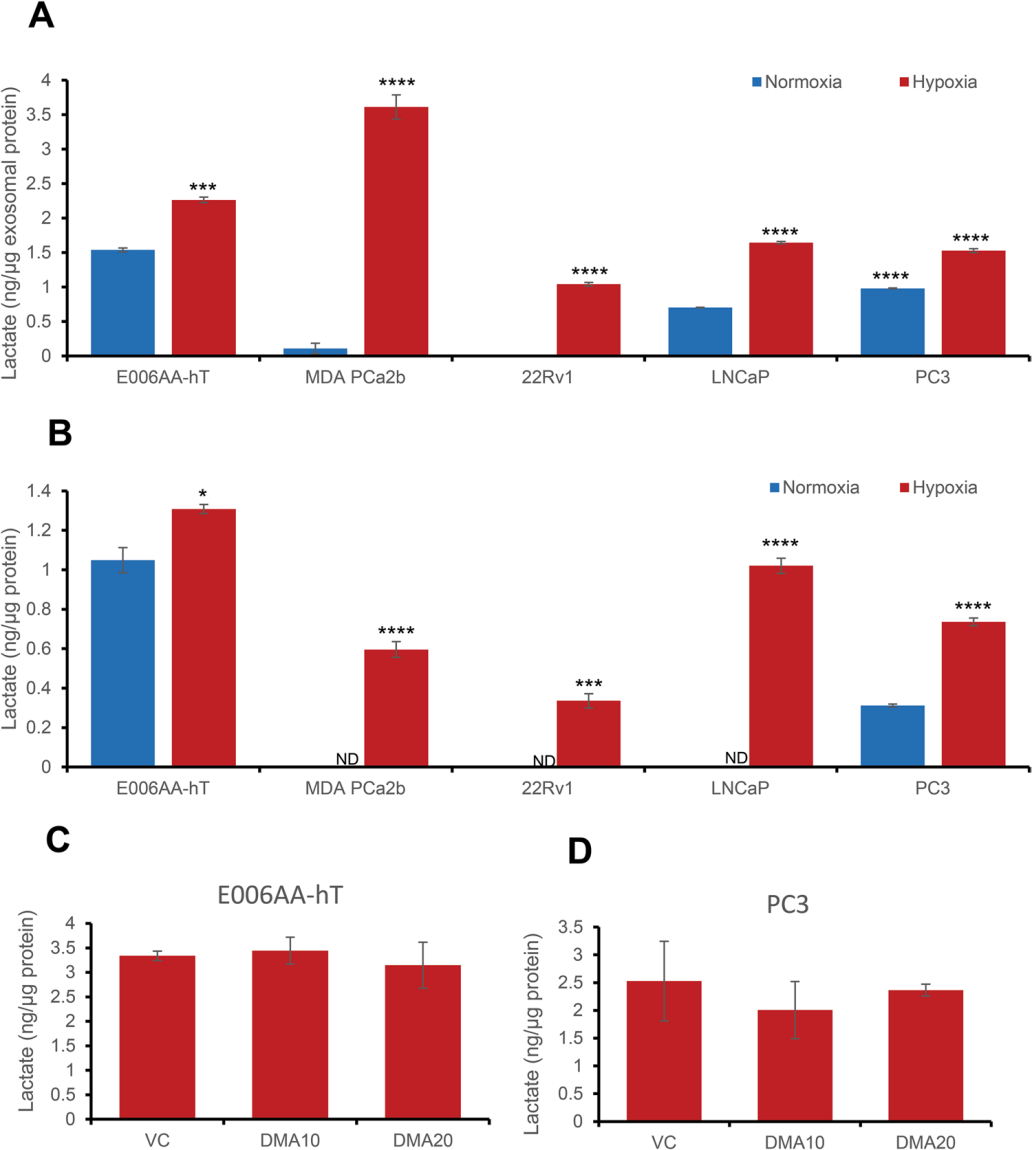
PCa cells accumulate and secrete higher amount of lactate loaded in exosomes under hypoxic conditions. (**A**) Exosomes were isolated from conditioned media of PCa cells cultured under normoxic (21% O_2_) or hypoxic (1% O_2_) conditions for 48 hrs by ExoQuick precipitation method; lactate levels in exosomes were measured using a lactate assay kit. (**B**) Cell lysates were prepared from PCa cells cultured under normoxic (21% O_2_) or hypoxic (1% O_2_) conditions for 48 hrs and lactate levels were measured. (**C**,**D**) E006AA-hT and PC3 cells were treated with DMA (10–20 µg/ml) and cultured under hypoxic (1% O_2_) conditions for 48 hrs. At the end, lactate levels were measured. In each case, values were normalized with protein concentrations and expressed as ng lactate/µg protein. Data represent mean ± SE of three replicates. *p < 0.05, ***p < 0.001, ****p < 0.0001.

Since exosome secretion offers a survival advantage to cells, we surmised that exosomes might remove metabolic waste. Lactic acid is a major metabolic end-product in cells under hypoxia. We measured lactate levels in exosomes secreted by PCa cells (E006AA-hT, MDA PCa 2b, 22Rv1, LNCaP, and PC3). Significantly higher amount of lactate was loaded in exosomes secreted by all cell lines under hypoxia compared to normoxia (Fig. 8A). Similar to exosomes, we observed higher lactate levels in all cell lines under hypoxia compared to normoxia (Fig. 8B).

Hypothesizing that lactate secretion might improve survival of PCa cells; we treated PCa cells (E006AA-hT and PC3) with the exosome biogenesis inhibitor DMA under hypoxia, and measured intracellular levels of lactate. However, these did not change significantly (Fig. 8C,D).

## Discussion

African American men in the US have higher incidence and deaths due to PCa compared to other races. To reduce or eliminate this health disparity, we must better understand the biology of PCa in African Americans. Thus, we compared exosome secretion by African American and Caucasian PCa cells under conditions of hypoxia, which is a key determinant of cancer aggressiveness^15,30^. We found that: (a) under hypoxia, both African American and Caucasian PCa cells secrete higher numbers of exosomes (more so in African American PCa cells), which offers a survival advantage to cells; (b) exosome biogenesis inhibitors could reduce the growth of PCa cells; and (c) hypoxic PCa exosomes have higher amount of lactic acid.

Exosome secretion increased under hypoxia in all PCa cell lines. King *et al*. earlier reported that under hypoxia, breast cancer cells produce higher amount of exosomes, dependent upon HIF-1α expression^25^. In another study, hypoxia-resistant multiple myeloma cells generated higher amount of exosomes than parental cells under acute hypoxic or normoxic conditions^31^. Parolini *et al*. suggested that an acidic microenvironment under hypoxia could cause higher exosome release and uptake by cancer cells^32^.

Several studies have shown that exosomes secreted under hypoxia offer growth advantages and promote cancer progression. For example, exosomes derived from hypoxic glioblastoma cells strongly induced angiogenesis^33^. Exosomes secreted by leukemia cells under hypoxia promoted the tube formation in human umbilical vein endothelial cells via miR-210^34^. Recently, we described that under hypoxia, PCa cells secrete exosomes that induce EMT, invasiveness, and stemness in naïve PCa cells, and promote a CAF-type phenotype in prostate fibroblasts^23^. Exosomes secreted by oral squamous cell carcinoma cells under hypoxia elicited a pro-metastatic phenotype through delivering miR-21 to normoxic cells^35^. Similarly, hypoxic exosomes promoted the bladder tumor growth via transferring long non-coding RNA-UCA1^36^. Recently, Hsu *et al*. reported that exosomal micro RNA from hypoxic cancer cells increases permeability and angiogenesis^37^. Together, these studies suggest that exosomes secreted under hypoxia promote tumor growth and progression. Since we observed that almost all African American PCa cells secrete more exosomes under hypoxia compared to Caucasian PCa cells, this could be the one reason for PCa aggressiveness in African Americans.

The role of lactate in tumor metabolism, tumor immunology, and remodeling of tumor microenvironment has been studied by several groups^38^. Lactic acid released by cancer cells increases acidification of the tumor microenvironment and promotes cancer progression. Further, lactic acid is considered a critical immunosuppressive metabolite^39^. Lactate inhibited the monocytes differentiation to dendritic cells and blocked cytokine release from dendritic cells^40^ as well as cytotoxic T cells^41^. Similarly, lactate enhanced the survival of regulatory T lymphocytes (Treg) cells and diminished the production of cytotoxic cytokines produced by CD8+T-cells^39^. Lactate is also emerging as a possible promotor of M2 polarization of macrophages, which generates an immune-tolerant environment and promote tumor growth and metastasis^42^.

Goetze *et al*. showed that lactate treatment increases the random migration of head and neck carcinoma cell lines^43^. Lactate exposure in cultured fibroblasts increased the hyaluronan production, and led to a higher expression of CD44, a predominant hyaluronan receptor on cell surfaces^44^. This is interesting observation as tumor stroma generally has higher hyaluronan levels produced by CAFs, which promotes growth and motility of cancer cells^45,46^. Lactate accumulation in primary tumors has clinical relevance as high lactate accumulation in primary cervical cancer predicted metastatic spread, tumor recurrence, and limited patient survival^47^. Here, we report the presence of high lactate in exosomes secreted by PCa cells under hypoxia, which could potentially cause both local and distant remodeling of the tumor microenvironment.

By secreting exosomes, cancer cells support tumor progression and metastasis by modifying the local and systemic micro-environment; therefore, targeting exosome biogenesis in cancer cells constitutes a novel cancer therapy^48^. Several investigators have reported that exosome biogenesis inhibitors such as GW4869 and DMA inhibit cancer growth. Kosaka *et al*. reported cell growth inhibition following inhibition of neutral sphingomyelinase 2 by GW4869^49^. Importantly, sphingomyelinase 2 controls the ceramide biosynthesis, which triggers the inward budding of exosomes from the endosomal limiting membrane^50^. GW4869 treatment in lung cancer-bearing mice significantly reduced the lung metastases^51^. Treatment of gemcitabine-exposed CAFs with GW4869 significantly reduced the survival in co-cultured epithelial cells^52^. Similarly, in colorectal cancer cells, inhibiting the secretion of exosomes in CAFs by GW4869 decreased the clonogenicity and tumor growth^53^. Pancreatic cancer cell-derived exosomes promoted chemoresistance and migration of surrounding cancer cells, which was mitigated by GW4869 treatment^54^. Recently, Matsumoto *et al*. reported that GW4869-induced inhibition of exosome secretion decreased the proliferation of B16BL6 cells. In the same study, intra-tumoral injection of GW4869 suppressed the tumor growth in mice^55^.

Similar to GW4869, several studies have shown that treatment with DMA inhibits growth of cancer cells. DMA targets the H^+^/Na^+^ and Na^+^/Ca^2+^ channels, which control intracellular Ca^2+^ concentrations (a regulator of exosome secretion)^56^. In mice bearing CT26 tumors, blocking these ion channels with DMA reduced the exosomes secretion^57^. Further, DMA treatment inhibited the tumor growth and decreased the proliferation of tumor cells in H6 hepatomas^58^. Another report suggested that DMA has a potent effect against peritoneal metastasis in gastric cancer^59^. Recently, Rojas *et al*. described anti-myeloma activity of DMA and provided a mechanistic rationale for its use as an alternative treatment option for patients with relapsed multiple myeloma^60^. Zheng *et al*. suggested that DMA sensitizes human pancreatic cancer cells to erlotinib through inhibiting the PI3K/Akt pathway^61^. Guan *et al*. suggested that inhibition of Na^+^/H^+^ exchanger-1 expression by NHE-1 specific shRNA or DMA decreased the viability and induced apoptosis in esophageal cancer cells^62^.

In accordance with the above studies, here we observed a significant decrease in colony-forming ability of both African American and Caucasian PCa cells following treatment with GW4869 and DMA. These compounds offer a novel therapeutic strategy against PCa and suggest the need for the development of new exosome biogenesis inhibitors. In this regard, recently, Datta *et al*. screened a drug library and identified Manumycin-A as a candidate that could inhibit exosome biogenesis and secretion in CRPC cells^63^. Cancer-specific pathways regulating exosome biogenesis need to be identified and targeted to control cancer growth and progression.

Overall, the present study found that African American and Caucasian PCa cells release higher amount of exosomes under hypoxia. Further, exosome secretion offers a survival advantage to PCa cells probably through removal of certain metabolites such as lactic acid. In addition, the present study highlights the potential value of targeting the exosome biogenesis pathway as a novel anti-cancer therapy.

## Materials and Methods

### Cell lines and reagents

Human PCa LNCaP, 22Rv1, PC3, and PWR-1E cells were purchased from ATCC (Manassas, VA). MDA PCa 2b cells were from Dr. Balaji (Wake Forest School of Medicine). RC77T/E and RC77N/E cell lines characterization was reported by Theodore *et al*.^64^ E006AA-hT cells were from Dr. Koochekpour (Roswell Park Memorial Institute). ExoQuick™ Kit was from System Biosciences (Mountain View, CA). CD63 (#ab59479), actin (#ab8226), and Rab5 (#ab18211) antibodies were from Abcam (Cambridge, MA). Secondary antibodies anti-rabbit IgG (H + L) F(ab’)2 fragment (Alexa Fluor® 555 Conjugate) (#4413 S), anti-mouse IgG (H + L) F(ab’)2 fragment (Alexa Fluor® 647 Conjugate) (#4410 S), and anti-mouse IgG (H + L) F(ab’)2 fragment (Alexa Fluor® 488 Conjugate) (#4408 S) were from Cell Signaling Technology (Danvers, MA). RPMI1640, penicillin and streptomycin, 0.25% trypsin, fetal bovine serum (FBS), and heat-inactivated FBS were from Gibco Laboratories (Gaithersburg, MD). Halt™ Protease and Phosphatase Inhibitor Cocktail, Keratinocyte-SFM and BRFF-HPC1™ media were from Thermo Fisher Scientific (Waltham, MA). DC™ Protein Assay kit was from Bio-Rad (Hercules, CA). The Poly- L-Lysine, Lactate Assay Kit II, 5-(N,N-Dimethyl)amiloride hydrochloride (DMA) and GW4869 were from Sigma-Aldrich (St. Louis, MO).

### Cell culture and hypoxia exposure

E006AA-hT, LNCaP, and 22Rv1 cells were grown in RPMI1640 medium supplemented with 10% FBS, 100 U/ml penicillin G and 100 µg/ml streptomycin sulfate. PC3 cells were grown in similar media as above but with 10% heat-inactivated FBS instead of FBS. RC77T/E, RC77N/E, and PWR1E cells were cultured in Keratinocyte SFM media supplemented with EGF (5 ng/ml) and bovine pituitary extract (0.05 mg/ml). MDA PCa 2b cells were cultured in BRFF-HPC1™ media on Poly-L-Lysine (50 µg/ml DPBS) coated flasks/plates. For normoxic (21% O_2_) condition, cells were cultured at 37 °C in a 5% CO_2_ humidified environment as an adherent monolayer. Hypoxia experiments were performed in a hypoxia chamber (Baker Ruskinn INVIVO_2_ 400 or BioSpherix X3 Xvivo system) at 1% O_2_ at 37 °C in a 5% CO_2_ humidified environment.

### Exosome isolation

Cells were cultured in exosome-depleted media under either normoxia (21% O_2_) or hypoxia (1% O_2_). After 48 hrs, conditioned media was collected and exosomes isolated by ultracentrifugation method, as reported by us previously^23,24^. In brief, the collected cell culture media was centrifuged at 300 g at 4 °C for 10 minutes; supernatant filtered through 0.22 µm filters (Merck Millipore); and filtrate concentrated using concentrators (150 K MWCO/20 ml, Thermo Scientific). The supernatants were then subjected to ultracentrifugation at 100,000 g for 90 min (L-80 Ultracentrifuge, 70.1 Ti fixed angel rotor, Beckman Coulter). Finally, the pelleted exosomes were suspended in DPBS. As indicated, exosomes were also isolated using a commercially available ExoQuick™ reagent (System Biosciences) according to the vendor’s protocol.

### Nanoparticle tracking analyses (NTA)

Exosome size distribution and concentrations were analyzed using a Nano sight LM10 system (Nano sight Ltd, Navato, CA) as reported by us earlier^23,24^. In each case, exosome concentration was normalized with corresponding cell number.

### Transmission electron microscopy (TEM)

A 400 mesh Formvar^®^/carbon-coated copper grid was soaked in 100% ethanol for 1 min. A 20 µl droplet of exosome samples (in PBS) was applied on a sheet of Parafilm. Then the grid was floated with the dark-coated side of the grid facing the sample and incubated for 90 minutes. Residual sample was washed from the grid by dipping it into 20 µl drops of molecular biology-grade water three times. Then grids were stained for 1 minute with 2% uranyl acetate. After allowing 60 minutes for drying, the grids were imaged with a TecnaiTM G2 Spirit BIOTWIN TEM equipped with an AMT Image capture 2Vu camera system.

### MTT assays

~2,000 cells were plated in a 96-well plate, followed by exposure to GW4869 under normoxia or hypoxia. At the end, MTT (5 mg/mL in PBS) was added for 2 hrs and then formazone formed in each well was dissolved in DMSO. Finally, absorbance was measured at 550 and 660 nm.

### Clonogenic assays

PCa cells (1,000 cells per well) were plated in 6-well plates, treated with exosome biogenesis inhibitors (DMA or GW 4869) and cultured either under normoxia or hypoxia for 7–8 days. Cells were then fixed with 10% formalin, stained with crystal violet solution, and then washed with deionized water. Plates were dried, scanned to obtain images and colonies (≥50 cells) were scored.

### Lactic acid measurements

Lysates (cells or exosomes) were prepared in RIPA buffer supplemented with Halt Protease and Phosphatase Inhibitor. Lactate levels were determined using a lactate assay kit II (Sigma-Aldrich, MAK065) following vendor’s protocol.

### Immunofluorescence

PCa cells were cultured in chamber slides (Nunc™ Lab-Tek™ II Chamber Slide™ System) under normoxia or hypoxia for 48 hrs. Thereafter, cells were fixed, permeabilized, and incubated overnight with primary antibodies (1:500; CD63, actin, and Rab5). Cells were then incubated with secondary anti-rabbit and/or anti-mouse antibodies conjugated with Alexa Fluor, followed by staining with Prolong® Gold Antifade Reagent with DAPI. Fluorescent images were captured using an Olympus FV1200 SPECTRAL Laser scanning Confocal Microscope at 60× objective lens (Olympus IX83 inverted platform).

### Statistical analyses

Statistical analyses were performed using Graphpad Prism 7.0 software (La Jolla, CA) by parametric unpaired t- tests. Significance was considered at p < 0.05.

### Data availability

The datasets generated during and/or analyzed during the current study are available from the corresponding author on reasonable request.
