# Author Correction: Hypoxia-induced exosome secretion promotes survival of African-American and Caucasian prostate cancer cells

**DOI:** 10.1038/s41598-018-24997-6

**Published:** 2018-04-24

**Authors:** Gati K. Panigrahi, Prakash P. Praharaj, Taylor C. Peak, Jessica Long, Ravi Singh, Johng S. Rhim, Zakaria Y. Abd Elmageed, Gagan Deep

**Affiliations:** 1 Department of Cancer Biology, Wake Forest School of Medicine, Winston-Salem, North Carolina USA; 2 Wake Forest Baptist Comprehensive Cancer Center, Wake Forest School of Medicine, Winston-Salem, North Carolina USA; 3 Center for Prostate Disease Research, Department of Surgery, Uniformed Services University of Health Sciences, Bethesda, MD USA; 4 Department of Pharmaceutical Sciences, Texas A&M Rangel College of Pharmacy, College Station, Texas USA

Correction to: *Scientific Reports* 10.1038/s41598-018-22068-4, published online 01 March 2018

In the original version of this Article, the author Zakaria Y. Abd Elmageed was incorrectly indexed. This error has now been corrected.

